# Modified trapdoor procedures using autogenous tricortical iliac graft without preserving the broken cartilage for treatment of osteonecrosis of the femoral head: a prospective cohort study with historical controls

**DOI:** 10.1186/s13018-020-01691-w

**Published:** 2020-05-24

**Authors:** Qi Cheng, Feng-chao Zhao, Shi-zhuang Xu, Li Zheng, Xin Zheng

**Affiliations:** 1 Department of Orthopedic Surgery, The Xuzhou Third Hospital, Xuzhou City, 221000 Jiangsu Province China; 2 Department of Orthopedic Surgery, the First Affiliated Hospital, College of Medicine, Zhejiang University, Hangzhou City, 310003 Zhejiang Province China; 3 Department of Orthopedic Surgery, The Affiliated Hospital of Xuzhou Medical University, Xuzhou City, 221002 Jiangsu Province China

**Keywords:** Osteonecrosis, Hip, Bone graft, Surgical procedure, Cartilage, Outcome

## Abstract

**Background:**

The aim of the present study was to investigate clinical and radiological outcomes of autologous tricortical iliac grafting performed through a window created at the femoral head without suturing the opened articular cartilage for the treatment of osteonecrosis of the femoral head (ONFH), called modified trapdoor procedures.

**Materials and methods:**

A total of 59 consecutive patients (67 hips; 36 males and 23 females) with ONFH were included in this study, which was conducted from April 2009 to March 2012. Patients’ age ranged from 27 to 46 years old, with a mean age of 36.3 years. Harris hip scores (HHS) were used to evaluate hip function pre- and postoperatively. Anteroposterior and frog-position X-rays and magnetic resonance imaging (MRI) were conducted to assess lesion location, size, and ARCO stage. Clinical failure was defined as score < 80 points or treatment by total hip arthroplasty (THA). Radiographic failure was defined as a > 3 mm of collapse in the hip. This group was retrospectively matched according to the ARCO stage, extent, location, etiology of the lesion, average age, gender, and preoperative Harris hip score to a group of 59 patients (67 hips) who underwent the “light bulb” approach between March 2007 and April 2009.

**Results:**

Mean follow-up was 91.2 ± 13.6 months (range, 75–115 months). Mean HHS was 91.3 ± 4.5, compared with 83.1 ± 4.5 in the “light bulb” cohort at the 6-year follow-up examination (*P* < 0.001). At the 6-year follow-up, for modified trapdoor procedures, five hips (8.5%) were classified as clinical failure, and three hips underwent total hip arthroplasty; seven hips were classified as (10.4%) radiographic failure. The clinical and radiographic failure of the hips treated with the modified trapdoor procedure was significantly lower compared to the hips treated with the “light bulb” procedure (*P* < 0.05). Survival of the joint was not significantly related to the location of the femoral head lesion between two groups; however, better clinical and radiographic results were observed in modified trapdoor procedures with size C and the ARCO stage III.

**Conclusion:**

The present study demonstrated superior midterm clinical results in ONFH with the use of autologous tricortical iliac block graft through a femoral head window, without suturing the opened articular cartilage. The femoral head-preserving procedure was superior compared to the “light bulb” procedure treatment in patients with postcollapse osteonecrosis and large lesion.

## Introduction

Untreated osteonecrosis of the femoral head (ONFH) has been associated with poor outcomes due to subchondral collapse and subsequent osteoarthrosis [1–5]. Studies in various populations have shown that the collapse of the femoral head occurs in 44%–79% of cases. The time interval between collapse and the ONFH diagnosis is usually less than 2 years [3–5]. Unfortunately, this disease usually affects relatively young and active patients: among cases associated with corticosteroid use, the mean age is 35 years; among cases associated with alcohol abuse, the mean age is 41 years; among cases associated with trauma, the mean age is 41 years; among cases of idiopathic etiology, the mean age is 40 years [6]. Total hip arthroplasty (THA) is often performed to treat this intractable disease (88%), with good short-term results [5]. Previous studies have shown that long-term outcomes for THA in the treatment of ONFH are inferior to those achieved for THA in the treatment of osteoarthritis [7, 8]. Although arthroplasty prosthesis designs and procedures have improved over recent decades, these patients are likely to undergo multiple revisions because of high levels of physical activity and long life expectancy. Previous studies have reported on the occurrence of complications such as progressive stem loosening and central migration of the prosthesis, which require revision surgery that is often more difficult, costly, and less effective than the original surgery, sometimes resulting in complications and/or mortality [9–11].

Therefore, there is an increasing need for joint-preserving strategies [5]. Multiple treatment modalities have been developed to avoid femoral head collapse and subsequent destruction of the hip joint or deferral of THA. The most commonly performed treatment procedures include core decompression, osteotomy, non-vascularized or vascularized bone graft, enriched bone substitutes, transplantation of a tantalum rod, and autologous bone marrow stem cell implantation. All these procedures aim to promote bone regeneration and/or provide mechanical support [10–26]. Yet, none of these treatment modalities have shown to be universally successful. The reasons for the failure of these treatments are that dead bone cannot be replaced by viable bone; subchondral bone does not have sufficient mechanical support and subsequent progression of osteoarthritis [27–31].

Most of the head-preserving procedures tried to maintain the articular cartilage over the necrotic zone, such as trapdoor procedure, light bulb procedure, Phemister technique, implantation of tantalum, etc. [5, 11–31]. Essentially, the necrotic subchondral bone is hard to heal with bone graft and marked collapsed still observed during conversion to THA [18]. The segmented collapsed cartilage may be combined with subchondral bone into a floating body, which inevitably results in pain. Numerous studies have shown that if the mechanical force is stable, the hips should show no symptoms or just slight discomfort. Partial defects of joint cartilage are not associated with poor clinical results [32, 33]. In one study with a minimum of 5 years of follow-up, the femoral head with chondral lesion did not result in worse clinical outcomes [34]. All of these findings indicate that mechanical failure, rather than cartilage degeneration, is the main cause of the symptom in ONFH without arthritis. Accordingly, we modified the trapdoor procedure by grafting an autologous tricortical iliac bone block through a window in the femoral head without preserving the articular cartilage. Herein, we described the principles and surgical techniques used to perform the procedure and compare the results achieved with the light bulb procedure.

## Materials and methods

### Patients

A total of 61 consecutive ONFH cases (69 hips) of patients who underwent bone grafting through a window in the femoral head between April 2009 and March 2012 were prospectively investigated. The diagnostic criteria for ONFH, as identified with magnetic resonance imaging (MRI) were a low signal band in the T1-weighted image and a high signal band in the corresponding STIR sequence [35]. The inclusion criteria were (1) consent to participate in the present study with at least 6 years of follow-up, (2) age ≤ 50 years, (3) no joint-space narrowing, (4) discomfort (pain in the hip, groin, buttock, or knee) that interfered with daily activity, (5) lack of a neurological disorder that could affect the source of the patient’s complaint, (6) no active connective tissue disease (e.g., rheumatoid arthritis, systemic lupus erythematosus), and (7) ARCO stages II and III. Two patients (2 hips) were lost to follow-up and thus were excluded from the study. Ultimately, 59 patients (23 women and 36 men, 67 hips) were included in the analysis. The age of these patients ranged from 27 to 46 years old, with a mean age of 36.3 ± 5.3 years. The etiology of osteonecrosis was as follows: the use of corticosteroids in 20 patients, alcohol abuse in 37 patients, and idiopathic etiology in 10 patients.

All hips were graded according to guidelines provided by the Association Research Circulation Osseous (ARCO) [36]. Lesions that occupied < 15% of the femoral head were classified as size A; lesions that occupied 15–30% of the femoral head were classified as size B; lesions that occupied > 30% of the femoral head were classified as C [36]. The location of the lesion on coronal midsection T1-weighted images was classified as one of four types, according to the criteria proposed by Sugano et al. [37]. Type A accounted for one third or less of lesions affecting the medial weight-bearing portion. Type B accounted for two thirds or less. Type C1 accounted for more than two thirds without extending laterally to the acetabular edge. Type C2 accounted for more than two thirds and extended laterally to the acetabular edge.

A total of 63 patients (72 hips), who underwent auto-iliac bone-grafting through a window at the femoral head–neck junction known as the “light bulb” approach for the treatment of osteonecrosis of the femoral head between March 2007 and April 2009, were retrospectively matched to 59 patients (67 hips) who underwent bone grafting through a window in the femoral head. The matching was based on the stage, extent, location, etiology of the lesion, average age, gender, and preoperative Harris hip score (Table 1).

**Table 1:** Demographic data for the two groups

Variables	The modified trapdoor group (*N* = 67)	The “light bulb” group (*N* = 72)	*P* value
Age (year)	36.3 ± 5.3	38.5 ± 4.1	0.894
Male:female	41:26	43:29	
Mean preop. Harris hip score (points)	67.3 ± 7.5	68.2 ± 6.3	0.910
Etiology (no. of hips)			0.946
Alcohol use	37	38	
Use of corticosteroids	20	22	
Idiopathic etiology	10	12	
Preoperative stage			0.685
ACRO II	45	46	
ARCO III	22	26	
Size			0.982
B	25	27	
C	42	45	
Location			0.916
B	12	11	
C1	29	32	
C2	26	29	

All cases were followed at 3, 6, and 12 months and then on an annual basis. Harris hip scores (HHS) and ARCO stage were recorded at each follow-up examination. Anteroposterior and frog-position X-rays, computed tomography (CT), as well as MRI scans were obtained.

HHS was used to evaluate clinical outcomes. Excellent, good, fair, and poor results were defined as > 90, 80–89, 70–79, and < 70, respectively. Scores < 80 points or patients who underwent THA were classified as examples of clinical failure. Clinical success was defined as a score of ≥ 80 points.

Each patient was also radiographically evaluated in terms of the progression in terms of the ARCO stage. Hips with > 3 mm of collapse or progress to ARCO IV were defined as radiographical failure [26].

Approval was obtained from the institutional review board, and written informed consent was obtained from each patient.

### Surgical technique

**Fig. 1:**
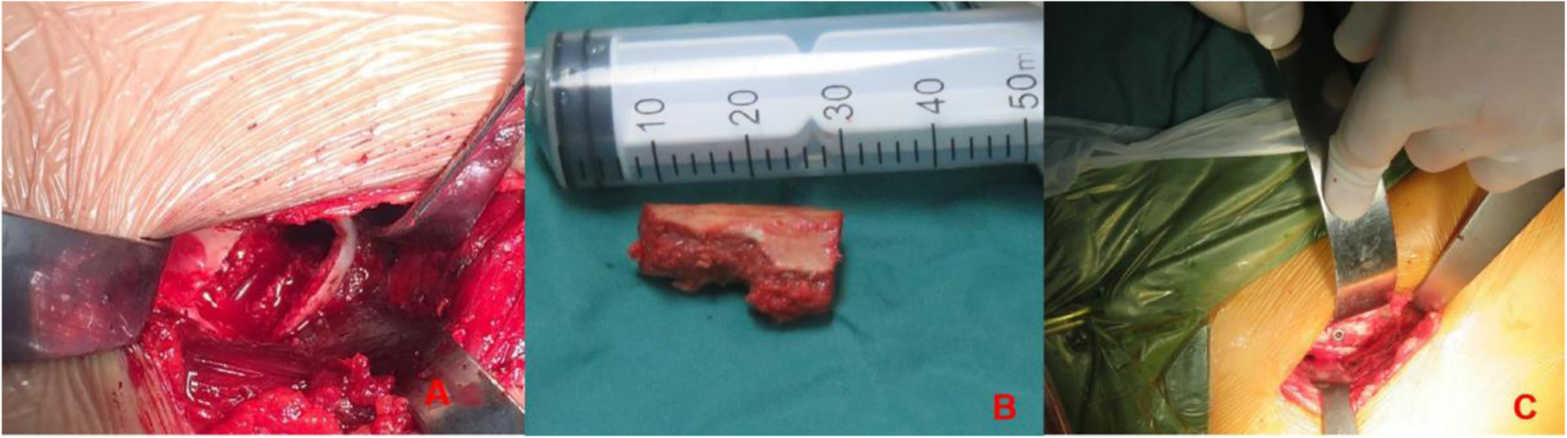
Bone grafting through a femoral head window. **a** Exposure of the femoral head without dislocation and creation of a cortical window in the femoral head and removal of all visible necrotic bone. **b** Autogenous iliac crest struts trimmed into its optimum shape. **c** Placement of a tricortical iliac bone graft in the groove and fixation with a screw

**Fig. 2:**
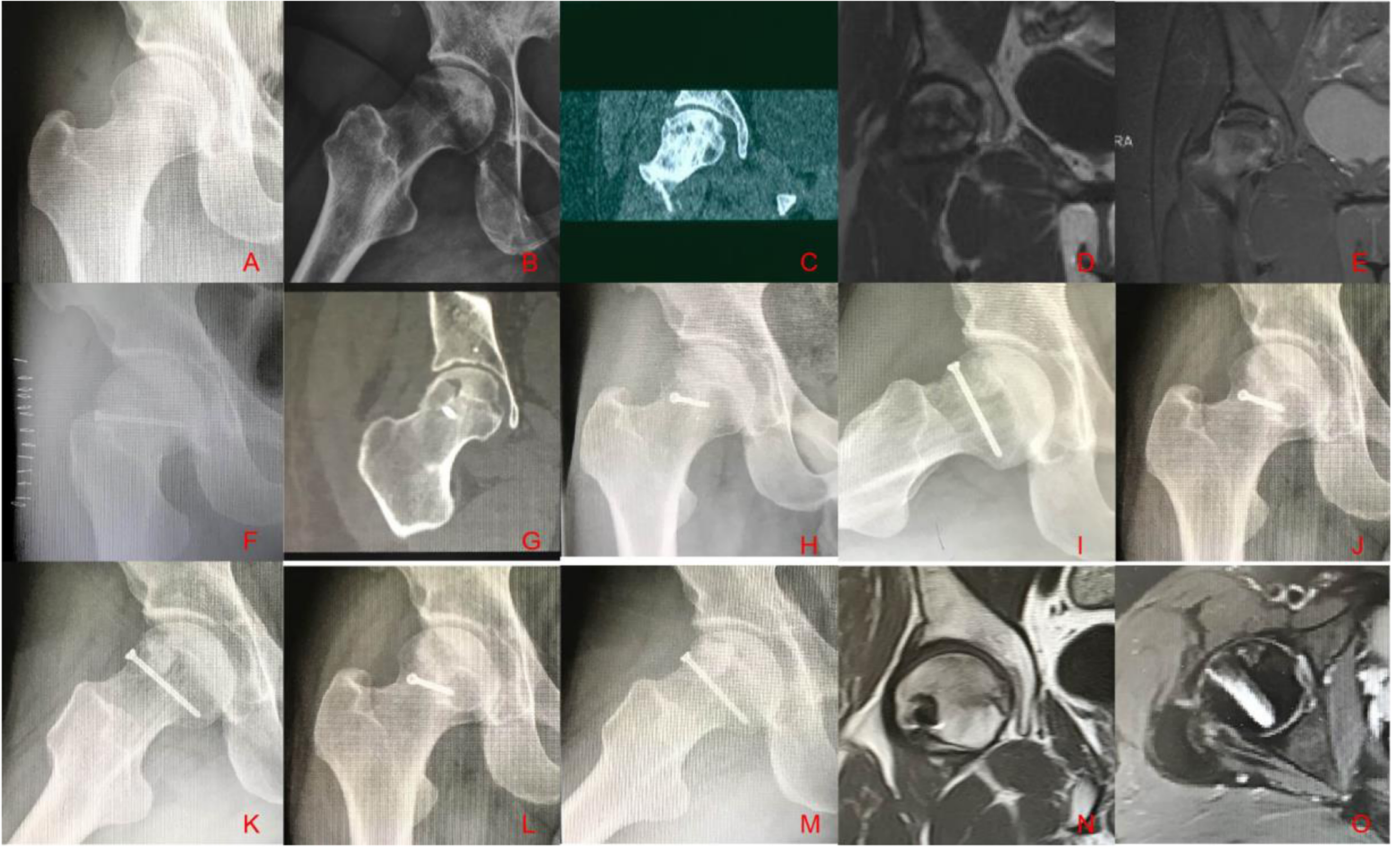
A 32-year-old man with osteonecrosis of the femoral head was treated with our modified trapdoor procedure. Anterior–posterior X-ray (**a**) and frog-position X-ray (**b**) show femoral head necrosis with segmental collapse. Coronal CT confirmed ONFH with collapse (**c**). Coronal T1 (**d**) and STIR (**e**) showed ONFH with edema. Postoperative radiography (**f**) showed necrotic bone that had been curetted and replaced with tricortical iliac block graft. Coronal CT (**g**) showed that necrotic bone had been curetted and replaced with a tricortical iliac block graft. The graft was in accordance with the contour of the femoral head. Anterior–posterior X-ray (**h**) and frog-position X-ray (**i**) obtained 1 year postoperatively show that the graft had healed to the host bone without collapse. Anterior–posterior X-ray (**j**) and frog-position X-ray (**k**) obtained 4 years postoperatively show that the graft had healed to the host bone, without collapse. Anterior–posterior X-ray (**l**) and frog-position X-ray (**m**) obtained 8 years postoperatively show that the contour of the femoral head was intact without collapse. Coronal T1 MR images (**n**) obtained 4 years postoperatively showed that the contour of the femoral head was intact, with the replacement of the necrotic bone by a viable bone, and normal cartilage at the femoral head. Axial STIR MR images (**o**) obtained 4 years postoperatively show that the contour of the femoral head remained intact; a portion of the necrotic bone has been replaced with a viable bone

Zhao et al. performed modified trapdoor procedures. The patient was put in a supine position, and the ilium was elevated (10–15°) by placing a sandbag under the buttock. The procedure was performed using an anterior minimally invasive approach and epidural or general anesthesia. An anterior straight incision, about 6 cm in length, was made about 1 cm distal and posterior to the anterior superior iliac spine. The hip joint capsule was exposed through the interval between the rectus femoris and the tensor fascia lata. The anterior part of the superficial aponeurosis was used to prevent damage to the lateral femoral cutaneous nerve. The femoral head was revealed by dissecting the joint capsule. MRI and computed tomography (CT) were preoperatively performed to localize the necrotic lesion and to determine the width of the tricortical bone block harvested from the iliac crest. The surgeon then created a window-like incision and used a scalpel to prepare a bone groove, extending from the head–neck junction to the acetabular rim, along the shaft of the femoral neck in the necrotic region of the femoral head. Necrotic bone was resected using osteotomes and power burrs, and curettage was performed until a bleeding surface was observed (Fig. 1). Ipsilateral autologous tricortical iliac bone was trimmed into the shape, which was consistent with the length of the window and the depth of the necrosis. Finally, the iliac bone block and small cancellous bone graft were tightly filled by hammering at the surgical site. This restored the prototype of the articular surface. The screws were used to fix bone graft through the iliac bone to the femoral head, and the screw heads were hidden in the bone. The resected segment of articular cartilage was no longer covered. The range of joint motion was measured, as well as the stability of the labrum. Any cam impingement was corrected. The joint capsule was then closed, and the procedure was completed with fascial, subcutaneous, and skin stitches. A drain was left in place for 24 h (Fig 2).

Weight-bearing was forbidden during the first 3 months, after which patients were allowed to begin partial weight-bearing. Full weight-bearing began at least 4 months postoperatively, depending on the extent of the clinical and radiological union.

### Statistical analysis

Continuous data are reported as means ± standard deviation. A paired t-test was used to compare changes in HHS. We used Pearson’s chi-squared or Fisher’s exact test to evaluate the relationship between variables and results. All analyses were performed with SPSS statistical software package, ver. 19.0 (SPSS, Inc., Chicago, IL, USA). *P* < 0.05 was considered to be statistically significant.

## Results

In total, 59 patients (67 hips) were followed for a minimum of 6 years (mean, 91.2 ± 13.6 months; range, 75–115 months). Average operation time (unilateral hip) was 50.5 ± 10.7 min, with a blood loss of 95.2 ± 22.8 ml.

The clinical and radiographic results are shown in Table 2. Mean HHS was 91.3 ± 4.5, compared with 83.1 ± 4.5 in the “light bulb” cohort at the 6-year follow-up examination (*P* < 0.001). The clinical results were good-to-excellent in 62 hips (92.5%), fair in 2 hips, and poor in 3 hips. At the 6-year follow-up examination, 7 hips treated with modified trapdoor procedures failed, according to the radiographic evaluation criteria. Five fair or poor hips progressed to collapse; three hips underwent further THA. Among the 72 hips with the light bulb procedure, 18 had radiographic signs of progression, and 15 of them had fair or poor clinical results. Nine hips further underwent THA because of the fair or poor results in the light bulb group. The clinical and radiographic failure of the hips treated with the modified trapdoor procedure was significantly lower compared to hips treated with the “light bulb” procedure (*P* < 0.05).

**Table 2:** Demographic results of the procedure

	Clinical failure rate		*P* value	Radiographical failure rate		*P* value
	The experimental group	The“light bulb” group		The experimental group	The “light bulb” group	
Preoperative stage						
ARCO II	1/45	3/46	0.617	2/45	3/46	1.000
ARCO III	4/22	12/26	0.041	5/22	15/26	0.014
Size						
B	1/25	2/27	1.000	1/25	3/27	0.611
C	4/42	13/45	0.023	6/42	15/45	0.038
Location						
B	0/12	2/11	0.217	0/12	3/11	0.093
C1	1/29	4/32	0.357	2/29	5/32	0.429
C2	4/26	9/29	0.173	5/26	10/29	0.205

When all hips in the two groups were compared at the time of the final follow-up, there was no significant difference between groups in terms of location (*P* > 0.05), while collapsed hips (ARCO stage III) were more prone to clinical and radiographic failure in the “light bulb” group (*P* = 0.041, 0.014). Furthermore, the rates of fair or poor clinical results and collapse for size C were significantly lower in the group treated with the modified trapdoor procedure (*P* = 0.023, 0.038).

There were no significant complications (e.g., fracture, joint infection, deep venous thrombosis) in any of the patients who underwent this procedure. Two patients with wound-related issues healed after their wounds were properly dressed.

## Discussion

The rationale for head-preserving procedures is a necessity to provide sufficient and long-lasting support to necrotic subchondral bone and cartilage [38] so as to prevent collapse and subsequent osteoarthrosis of the joint. In previous studies, a tantalum rod was used to provide direct mechanical support [16], while a bone graft was aimed to replace the dead bone with viable bone [12–14]. Various types of stem cells and/or biofactors were used to facilitate bone formation and remodeling, alone or in combination with other methods [15, 19]. Vascularized bone graft, which is technically demanding, is deemed to have better effect than nonvascularized bone graft. Nonvascularized bone graft procedures are currently the most popular methods for preserving the necrotic head and/or deferring THA. Following three surgical techniques are commonly used: (1) the Phemister technique (grafting via a core decompression track from the greater trochanteric area), (2) “light bulb” procedure (grafting through a femoral neck or femoral head–neck junction window, and (3) trapdoor procedure (grafting through a femoral head window).

Keizer et al. [21] described a cohort of cases that underwent autogenous cancellous bone grafting via a core tract (Phemister technique), after 7 years of mean follow-up, resulting in 34 of 78 hips (44%) that required additional surgery. This procedure is currently avoided since the necrotic bone cannot be sufficiently debrided, thus resulting in insufficient solid support for subchondral bone [17, 20]. In 1998, Mont et al. [18] introduced the trapdoor procedure, which involves creating a window at the femoral head. In that study, 20 out of 24 Ficat stage III hips (83%) had good or excellent outcomes. This procedure involves disrupting the integrity of the femoral head cartilage, which has been shown to prevent healing. More and more clinicians are choosing bone grafting through a femoral neck or head–neck junction window without disrupting the weight-bearing cartilage (light bulb procedure). Rosenwasser et al. [23] chiseled out a femoral head–neck junction window with a success rate of 81% at 12 years.

All of these procedures have the scope of maintaining the articular cartilage over the necrotic zone. In our study, the clinical survival rate of the procedure without suturing the opened articular cartilage was 92.5% for a minimum of 6 years, which was significantly superior to the light bulb procedure. The light bulb procedure did not damage the articular cartilage; however, it encountered two difficult technical problems. First, it is difficult to completely remove the necrotic bone. Actually, the necrotic subchondral bone is hard to heal with bone graft; marked collapse is still observed during conversion to THA [18, 27–31]. Second, we could not perform perfect bone grafting to fill the canal. Even with a sophisticated operation for the restoration of the spherical shape of the femoral head, some mild joint incongruence occurs, thus making it impossible to maintain the sphericity without collapse for longer periods. The collapsed cartilage and subchondral bone may serve as a floating body, thus leading to negative health outcomes. With partial cartilage defects, the hip function can still reach satisfactory levels. The previous study showed that femoral head with part chondral lesion detected during arthroscopic surgery still functioned well with minimum 5 years of follow-up [34]. These findings indicate that mechanical failure, rather than cartilage degeneration, is the leading cause of pain in most patients, especially in the early stages. Accordingly, we modified the trapdoor procedure. After thorough debridement, an autologous tricortical iliac block graft combined with morselized bone was implanted. Broken cartilage and necrotic subchondral bone were no longer replanted. Compared with previous studies [12–26], this report includes superior results. The rate of clinical success (good or excellent outcome) was 92.5% for 67 hips.

Larger lesions and more advanced linear collapse increase the relative risk of failure. In a retrospective study of 110 patients who underwent 138 light bulb procedures with a mean follow-up of 25.37 months, Wang and colleagues [22] demonstrated that the “light bulb” technique should not be performed once the subchondral collapse is present. Similarly, Sotereanos et al. [39] showed that the probability of conversion to total hip arthroplasty within an average of 5.5 years after autogenous grafts was 38% for stages III and IV hips. The rate of collapse for modified trapdoor procedures was only 18.2% or 9.5% among ONFH hips of ARCO III or size C. When compared with light bulb procedures, modified trapdoor procedure is also a sensible procedure for patients with postcollapse osteonecrosis. It does not involve the cartilage lesion of ARCO III and thoroughly removes dead bone tissue for size C. Autogenous tricortical iliac provides sufficient support, effectively fills necrotic cavity, and incorporates the graft to the recipient bone of the femoral head.

Compared with the traditional trapdoor procedure, the first advantage of our technique refers to a tricortical iliac block graft that is fixed with one or two screws, and thus can more easily heal with the host bone and provide good structural support [18, 32]. Even though the autogenous iliac crest transplantation is an effective treatment, there is a risk of bone flap loosening. Therefore, screw fixation is needed to increase the stability of the bone flap. The second advantage is that our procedure does not involve suturing of the opened articular cartilage, so there are no issues related to necrotic subchondral bone healing with graft bone or cartilage–cartilage interface healing. Floating cartilage slices, just as osteochondritis dissecans, may be another reason for unsatisfactory results. Third, the use of the anterior approach without dislocation of the hip leads to less damage to posterior structures, with excellent exposure and preservation of the blood supply [40, 41]. Finally, this procedure is minimally invasive, technically simple, short, leads to fewer complications, and associated with no adverse effect on the procedure of late arthroplasty.

## Conclusion

Our results suggest that the modified’ trapdoor procedure is associated with better clinical and radiographic results than the light bulb procedure in femoral head osteonecrosis, particularly in those with ARCO stage III or size C disease. Finally, in patients with postcollapse osteonecrosis, which tends to destroy articular cartilage and damage bone beneath the cartilage, the procedure appears to delay hip arthroplasty in the majority of patients, also eliminating the need for eventual arthroplasty for many of them.

## Data Availability

We do not wish to share our data to protect our patients’ privacy; also, the policy of our hospital prohibits the sharing of the data without permission.

## Abbreviations

ONFH: Osteonecrosis of the femoral head
THA: Total hip arthroplasty
CT: Computed tomography
